# Proteome Profiling—Pitfalls and Progress

**DOI:** 10.1002/1097-0061(20000630)17:2<81::AID-YEA22>3.0.CO;2-Z

**Published:** 2000

**Authors:** Paul A. Haynes, John R. Yates III

**Affiliations:** Novartis Agricultural Discovery Institute 3115 Merryfield Row San Diego CA 92121 USA

## Abstract

In this review we examine the current state of analytical methods in proteomics. The conventional methodology using two-dimensional electrophoresis gels and mass spectrometry is discussed, with particular reference to the advantages and shortcomings thereof. Two recently published methods which offer an alternative approach are presented and discussed, with emphasis on how they can provide information not available via two-dimensional gel electrophoresis. These two methods are the isotope-coded affinity tags approach of Gygi *et al*. and the two-dimensional liquid chromatography–tandem mass spectrometry approach as presented by Link *et al*. We conclude that both of these new techniques represent significant advances in analytical methodology for proteome analysis. Furthermore, we believe that in the future biological research will continue to be enhanced by the continuation of such developments in proteomic analytical technology.


**Yeast**
*Yeast* 2000; **17**: 81–87.

**Review Article**

### Proteome profiling—pitfalls and progress

Paul A. Haynes* and John R. Yates III
*Novartis Agricultural Discovery Institute, 3115 Merryfield Row, San Diego, CA 92121, USA*

*Correspondence to:
P. A. Haynes, Novartis Agricultural Discovery Institute,
3115 Merryfield Row, San Diego, CA 92121, USA.
E-mail:
p.haynes@nadii.novartis.com*

### Abstract

**In this review we examine the current state of analytical methods in proteomics. The conventional methodology using two-dimensional electrophoresis gels and mass spectrometry is discussed, with particular reference to the advantages and shortcomings thereof. Two recently published methods which offer an alternative approach are presented and discussed, with emphasis on how they can provide information not available via two-dimensional gel electrophoresis. These two methods are the isotope-coded affinity tags approach of Gygi *et al.* and the two-dimensional liquid chromatography–tandem mass spectrometry approach as presented by Link *et al.* We conclude that both of these new techniques represent significant advances in analytical methodology for proteome analysis. Furthermore, we believe that in the future biological research will continue to be enhanced by the continuation of such developments in proteomic analytical technology. Copyright © 2000 John Wiley & Sons, Ltd.**

**Keywords: two-dimensional electrophoresis; mass spectrometry; protein analysis; proteome; methods; liquid chromatography; liquid chromatography–mass spectrometry**

### Why do we analyse proteins?

The long-standing paradigm in biology is that DNA synthesizes RNA, which synthesizes protein. Conventional wisdom states that the blueprint for how to assemble a cell is contained in the genetic code, but it is important to realize that the bricks and mortar used in the building process are predominantly proteins. Thus, proteins are the molecules in cells that are directly responsible for maintenance of correct cellular function, and consequently the viability of the organism that contains the cells. In recent years, the simultaneous study of the whole range of proteins expressed in a cell at any given time has become an area of great interest. This has led to the classification of a new subdiscipline of protein chemistry known as 'proteomics', where a proteome is defined as the protein complement expressed by the genome of an organism or cell type [49].

The tools used in the analysis of proteins, however, still lag behind the analogous tools used in the analysis of DNA and RNA. It is relatively facile to undertake identification and quantification of many different DNA or RNA molecules in a single experiment using an array prepared from a single initial sample. This can be done using such techniques as DNA chips and cDNA microarrays [41,23], differential display PCR [24] and serial analysis of gene expression [46,47]. It is simply not possible to perform the same type of experiments at the protein level using two-dimensional gel electrophoresis (2DE), which is the current widely accepted technology in this area despite the fact that it suffers from several major shortcomings.

In discussing analytical methods to be used in proteome analysis, consideration must be given to the fact that the number of proteins expressed at one time in a given cellular system is typically in the thousands or tens of thousands. Any attempt to categorize and identify all of these proteins simultaneously must use methods which are as rapid as possible to enable completion of the project within a reasonable time frame. Thus, an idealized proteomics technology would consist of a combination of the following features: high sensitivity, high throughput, the ability to differentiate differentially modified proteins, and the ability to quantitatively display and analyse all the proteins present in a sample. In this review we will compare and contrast the current technology with two recently developed

Copyright © 2000 John Wiley & Sons, Ltd.

analytical methods that may, with further development, overcome several of the problems inherent in 2D gel electrophoresis.

### The 2D electrophoresis approach

The most commonly used technique in global proteome analysis is 2D electrophoresis using isoelectric focusing/sodium dodecyl sulfate–polyacrylamide gel electrophoresis (IEF/SDS–PAGE). In 2DE, proteins are first separated by isoelectric focusing (IEF) and then further resolved by SDS–PAGE in the second, perpendicular, dimension. Separated proteins are visualized by staining or autoradiography to produce a 2D array that can contain thousands of proteins [15,22].

The identification of individual proteins from polyacrylamide gels, of one or two dimensions, has traditionally been carried out using co-migration with known proteins [21], immunoblotting, N-terminal sequencing [3,28] or internal peptide sequencing [2,36]. In recent years there has been a fundamental shift in the ways such experiments are performed, principally due to the explosive growth of large-scale genomic databases. The current widely used method relies on excising spots from gels, proteolytically digesting the spots, and then extracting the peptides produced. The final stage involves analysing these peptides by mass spectrometry (MS) or tandem mass spectrometry (MS–MS) and then correlating the mass spectral data derived from the peptides with information contained in databases of protein sequence, genomic sequence or expressed sequence tags (ESTs) [11,27,51].

It is clear that this type of approach will become even more widely used in the near future as complete genome sequence data becomes available for more and more organisms. Complete genome sequences have already been reported for a number of organisms, among them *Haemophilus influenzae* [13], *Saccharomyces cerevisiae* [14], *Escherichia coli* [5], *Caenorhabditis elegans* [7] and *Drosophila melanogaster* [1]. The human genome project is, of course, one of the major driving forces in biomedical research in recent years. At the time of writing, the first reports have just been released that the first draft of the human genome sequence is now complete [43].

### The pros and cons of 2D electrophoresis

The disadvantages of 2D electrophoresis are that it is very time-consuming, essentially non-quantitative, does not work well for hydrophobic proteins, and has a limited dynamic range. Large-format gels typically require at least 24 h to complete, and for practical reasons are often completed over the course of several days. Staining of individual 2DE spots can be measured and compared using scanning densitometry, but there are so many caveats attached to the data that the results are of questionable value. Many staining techniques, such as silver staining, suffer from a limited dynamic range, so that the intensity of more abundant spots is not linearly correlated to that of less abundant spots. Moreover, some types of proteins, especially those that are post-translationally modified, can give qualitatively and quantitatively different staining when compared to similar amounts of other proteins. Hydrophobic proteins, particularly those of high molecular weight, are especially problematic in 2D gels because the presence of SDS is incompatible with successful first-dimension IEF. Thus, most IEF sample buffers solubilize a wide range of proteins by including high concentrations of chaotropic salts, such as urea, and lower levels of mild detergents, such as CHAPS. It should be noted, however, that significant progress in overcoming this particular limitation has been made in recent years, including the development of new detergents with greater solubilizing power [38,37], and the selective application of organic solvents to aid in solubilizing hydrophobic proteins [31].

Several studies have shown that the majority of proteins identified in 2DE are the more abundant and the more long-lived proteins in the cell. In a study of more than 150 proteins identified in 2DE of yeast cells, for example, no proteins were identified with a codon bias value of less than 0.1, an arbitrarily defined cut-off indicating low abundance [18]. In contrast, calculated values indicate that over half of the 6000 genes in yeast [14] have a codon bias index of less than 0.1 and thus are unlikely to be seen in 2DE without prior enrichment. Several techniques have been proposed as generic sample pretreatment strategies to increase the total number of spots that can be visualized in 2DE. These include sequential extraction of a sample with buffers of increasing solubilizing power, which generates fractions on the basis of

Copyright © 2000 John Wiley & Sons, Ltd. 

hydrophobicity [30], and using narrow-range pH gradients for the first dimension IEF, which expands the resolution in a given range [9,12].

Despite these disadvantages, 2DE remains the method of choice for displaying proteins as the front end of a proteomics project, for two main reasons: first, because it can be used to visualize a very large number of proteins simultaneously; and second, because it can be used in a differential display format. The ability to study complex biological systems in their entirety rather than as a multitude of individual components makes it far easier to discover the many complex relationships between proteins in functioning cells. This type of experiment, where the aim is to catalogue as many of the expressed proteins as possible and build up a database of expressed proteins, is often referred to as a 'proteome project'. Large-scale proteome characterization projects which have been reported include those of microorganisms such as *Saccharomyces cerevisiae* [20], *Escherichia coli* [45], *Haemophilus influenzae* [26], *Mycobacterium tuberculosis* [44], *Ochrobactrum anthropi* [48], *Salmonella enterica* [32], *Spiroplasma melliferum* [8], *Synechocystis* spp. [39], *Dictyostelium discoideum* [50] and *Rhizobium leguminosarum* [16], and tissues including human liver [4], human plasma [4], human fibroblasts [6], human keratinocytes [6], human bladder squamous cell carcinomas [6], mouse kidney [6] and rat serum [10,19,29].

Additionally, an ambitious attempt to undertake a complete human proteome project has recently been announced by the same research group responsible for one of the major successful efforts in the human genome project [40]. It remains to be seen whether this effort will be quite so successful. One major issue with establishing any proteome characterization project is defining the proteome in question. A single genome can give rise to an essentially infinite number of qualitatively and quantitatively different proteomes, depending on such variables as the stage of the cell cycle, growth and nutrient conditions, temperature and stress response, pathological conditions and strain differences, to name but a few. Another way of expressing the same problem is that genomes are essentially static, while proteomes are, by their very nature, dynamic and, therefore, a 2DE-based proteome project can only represent a snapshot rather than the whole constantly moving picture.

Although 2DE is not strictly quantitative, as noted above, the presence or absence of one or more spots in one gel when compared to another is readily detectable. Using this approach, the state of a cellular system in response to a particular treatment can be assessed using 2DE of samples of each state, which allows for the simultaneous assessment of the effect of the treatment on many proteins at once, rather than measuring, for example, levels of a single enzyme. This type of differential display experiment can be used to directly visualize the proteins which are affected during, for example, cell differentiation, gene knockouts, changes in growth or nutrient conditions, or treatment of cultured cells with a potential therapeutic drug. Once these protein spots are identified, knowledge of the proteins that are directly affected by a particular treatment can identify the biochemical pathways involved and so be of great value in deciding the direction of future research. Thus, in this implementation, proteome analysis is used as a biological assay, rather than as a database as described above.

In summary, despite numerous drawbacks and limitations, 2DE remains a powerful and versatile tool in proteome analysis. It is clear, however, that there is room for improvement in the efficiency of analysis, and this may be achieved by both incremental advances in current methods or development of new technologies.

### Isotope-coded affinity tag peptide labelling

The first of the new methodologies that we feel has the potential to have a great impact on proteome research is known as isotope-coded affinity tag (ICAT) peptide labelling [17]. This is an approach that combines accurate quantification and concurrent sequence identification of the individual proteins in complex mixtures. The method is based on a newly synthesized class of chemical reagents (ICATs) used in combination with tandem mass spectrometry. The ICAT reagent contains a biotin affinity tag and a thiol specific reactive group, which are joined by a spacer domain which is available in two forms; regular and isotopically heavy, which includes eight deuterium atoms.

In brief, the method consists of four steps. First, a reduced protein mixture representing one cell state

Copyright © 2000 John Wiley & Sons, Ltd.

is derivatized with the isotopically light version of the ICAT reagent, while the corresponding reduced protein mixture representing a second cell state is derivatized with the isotopically heavy version of the ICAT reagent. Second, the labelled samples are combined and proteolytically digested to produce peptide fragments. Third, the tagged cysteine-containing peptide fragments are isolated by avidin affinity chromatography. Finally, the isolated tagged peptides are separated and analysed by microcapillary tandem mass spectrometry, which provides both identification of the peptides by fragmentation in MS–MS mode and relative quantitation of labelled pairs by comparing signal intensities in MS mode. It should be noted that a method based on similar principles, which has the one crucial difference of being based on metabolic labelling of cells and is therefore limited to use with organisms which can be successfully cultured, has also been recently reported [33].

There are several advantages of this approach when compared to 2DE. There is no need to run time-consuming 2DE experiments, and the approach is scaleable so that, in theory, a large enough amount of sample can be used to enable analysis of low-abundance proteins. The method is based on stable isotope labelling of isolated protein samples, so it does not require the use of metabolic labelling or radioactivity. Most important of all, however, is that it provides accurate relative quantification of each peptide identified. For example, if a protein is present at the same level in the two original samples, the amount of each peptide detected will be the same. If, however, a protein is present at a 10-fold higher level in the sample derivatized with the heavy ICAT reagent, then the amount of heavy ICAT-labelled peptide detected will be 10 times greater than the amount of light ICAT-labelled peptide detected. It should be emphasized that, although quantitation by mass spectrometry is often unreliable, in this case the peptides act as mutual internal standards, since they are chemically identical and differ only by eight neutrons, and thereby eliminate potential problems due to differing ionization efficiencies or other physicochemical properties.

There are also several obvious disadvantages to this technique as it is currently presented, but all of them appear to be surmountable in the course of future development. The proteins must, first of all, contain cysteine, which is true for an estimated 92% of yeast proteins, for example, and those cysteines must also be flanked by appropriately spaced protease cleavage sites. Moreover, the ICAT tag is a large moiety when compared to the size of some small peptides and thus may interfere with peptide ionization and can greatly complicate mass spectral interpretation. All of these problems may be overcome by designing different reagents with specificity for other peptide side-chains, using a smaller tag group, and using different proteases.

In summary, it is sufficient to say that the published application example, involving the identification and quantification of galactose- and glucose-repressed proteins in yeast harvested under different growth conditions, represents a significant advance in proteome analysis. Not only were the proteins affected by different growth conditions unambiguously identified, but also their relative amounts were accurately quantified in the course of the same experiment. It is to be hoped that further research in this area will yield even more promising data in the future.

### Multi-dimensional protein identification technique

The second of the new methodologies that we believe represents a significant step forward in proteome analysis is the use of multidimensional liquid chromatography coupled to tandem mass spectrometry (LC–LC–MS/MS). The LC–LC–MS/MS method, as recently reported for use in the analysis of complex mixtures of peptides [25], is now commonly known by the acronym MudPIT, for multi-dimensional protein identification technique. This method has been previously reported in various incarnations, involving reversed phase columns coupled to either cation exchange columns [34] or size exclusion columns [35]. However, it was only when the technique was employed with a mixed-bed microcapillary column containing strong cation exchange (SCX) and reversed phase (RPC) resins that the true utility of this method was demonstrated [25]. This chromatographic method contains numerous steps, as outlined below.

First, a denatured and reduced protein mixture is digested with trypsin to produce peptide fragments. The mixture is loaded onto a microcapillary column containing SCX resin upstream of RPC resin, eluting directly into a tandem mass spectrometer.

Copyright © 2000 John Wiley & Sons, Ltd. 

A discrete fraction of the absorbed peptides are displaced from the SCX column onto the RPC column using a step gradient of salt, causing the peptides to be retained on the RPC column, while contaminating salts and buffers are washed through. Peptides are then eluted from the RPC column using an acetonitrile gradient, and analysed by MS/MS. This process is repeated using increasing salt concentration to displace additional fractions from the SCX column. This is applied in an iterative manner, typically involving 10–20 steps, and the MS/MS data from all of the fractions are analysed by database searching [11,51] and combined to give an overall picture of the protein components present in the initial sample.

There are several advantages of the MudPIT technique, beginning with the fact that it once again avoids the need for time-consuming 2DE and can, in fact, be run in a fully automated system. The use of two dimensions for chromatographic separation also greatly increases the number of peptides that can be identified from very complex mixtures. For example, analysis of a total yeast cell lysate identified 749 unique peptides, from 189 unique proteins, in a single MudPIT experiment [25], which is far more than would be expected from a conventional LC–MS/MS experiment. Perhaps the most important point, however, is that the method has a very wide dynamic range and none of the protein solubility problems associated with 2DE since the proteins are all proteolytically digested *en masse*. This is graphically demonstrated in the published example, where the technique was used to characterize whole protein complexes. The yeast ribosomal 80s complex was found to contain 64 proteins by analysis of 56 discrete spots visible in a 2DE experiment, but an additional 11 proteins were identified by analysing the same sample using MudPIT [25].

The main drawbacks of this approach are concerned with post-experimental analysis. The sheer volume of data collected in a MudPIT experiment consisting of 10–20 cycles of reversed-phase chromatography presents a significant problem in terms of both computing power required to complete database searching and the time required to collate and assemble the data into an understandable format. Moreover, the approach is generally limited to use with organisms that have complete genome sequence data available for searching. For the analysis of a single 2DE spot, it is possible to obtain *de novo* sequence data of peptides, using either software or manual interpretation or a combination of both. This is, however, a labour-intensive and time-consuming task that could not be practically applied to the number of tandem mass spectra collected in a typical MudPIT experiment.

These problems are all readily solvable, which makes this approach even more attractive. Computing resources continue to steadily increase in performance and become more affordable. Mass spectrometric instrumentation and *de novo* sequencing algorithms will surely improve, making *de novo* sequencing on a larger scale more practical. Additionally, the technique could be combined with some of the strategies used for improving success in MS–MS based on *de novo* sequencing experiments, such as employing proteolytic digestion in $^{18}O$ enriched water [42] to provide an isotopic end label. And, of course, at some point in the future, complete genomic sequence data will be available for all the major research organisms and therefore *de novo* sequencing will no longer be required.

In summary, MudPIT represents a viable alternative to 2DE for the analysis of certain complex mixtures. It is a technique that is best suited to rapidly building a proteomic database, rather than being applied in a differential display proteomic assay. This approach is clearly going to become increasingly attractive as a means of extracting as much information as possible in a short time from a protein sample that could represent a complex or even a relatively simple whole organism, particularly one for which large amounts of genomic sequence data are readily available.

### Conclusion

Identification and quantification of large numbers of proteins in as short a time as possible will become increasingly important in the future. As we enter the post-genomic era, the search for new enabling technologies will become ever more intense. In this review, we have tried to demonstrate that such techniques and methodologies are becoming available, and are now ready to be used in solving interesting and exciting biological problems.

Copyright © 2000 John Wiley & Sons, Ltd. 

### Abbreviations

2DE: two-dimensional electrophoresis; EST: expressed sequence tag; ICAT: isotope-coded affinity tag; IEF: isoelectric focusing; IPG: immobilized pH gradient; LC–MSMS: liquid chromatography–tandem mass spectrometry; LC–LC–MS/MS: liquid chromatography–liquid chromatography–tandem mass spectrometry; MS: mass spectrometry; MS/MS: tandem mass spectrometry; MudPIT: multi-dimensional protein identification technique; PAGE: polyacrylamide gel electrophoresis; PCR: polymerase chain reaction; RPC: reversed phase chromatography; SCX: strong cation exchange chromatography; SDS: sodium dodecyl sulphate; SDS–PAGE: sodium dodecyl sulphate–polyacrylamide gel electrophoresis

### Acknowledgements

The authors would like to thank Dave Schieltz and Jing Wei for thoughtful discussions, and acknowledge the continuing support and enthusiasm of Steve Briggs.

### References

1. Adams MD, Celniker SE, Holt RA, Evans CA, Gocayne JD, Amanatides PG, Scherer SE, Li PW, Hoskins RA, Galle RF, George RA, Lewis SE, Richards S, Ashburner M, Henderson SN, Sutton GG, Wortman JR, Yandell MD, Zhang Q, Chen LX, Brandon RC, Rogers YH, Blazej RG, Champe M, Pfeiffer BD, Wan KH, Doyle C, Baxter EG, Helt G, Nelson CR, Gabor Miklos GL, Abril JF, Agbayani A, An HJ, Andrews-Pfannkoch C, Baldwin D, Ballew RM, Basu A, Baxendale J, Bayraktaroglu L, Beasley EM, Beeson KY, Benos PV, Berman BP, Bhandari D, Bolshakov S, Borkova D, Botchan MR, Bouck J, Brokstein P, Brottier P, Burtis KC, Busam DA, Butler H, Cadieu E, Center A, Chandra I, Cherry JM, Cawley S, Dahlke C, Davenport LB, Davies P, de Pablos B, Delcher A, Deng Z, Mays AD, Dew I, Dietz SM, Dodson K, Doup LE, Downes M, Dugan-Rocha S, Dunkov BC, Dunn P, Durbin KJ, Evangelista CC, Ferraz C, Ferriera S, Fleischmann W, Fosler C, Gabrielian AE, Garg NS, Gelbart WM, Glasser K, Glodek A, Gong F, Gorrell JH, Gu Z, Guan P, Harris M, Harris NL, Harvey D, Heiman TJ, Hernandez JR, Houck J, Hostin D, Houston KA, Howland TJ, Wei MH, Ibegwam C, *et al.* 2000. *Science* **287**: 2185–2195.
2. Aebersold RH, Leavitt J, Saavedra RA, Hood LE, Kent SB. 1987. *Proc Natl Acad Sci U S A* **84**: 6970–6974.
3. Aebersold RH, Teplow DB, Hood LE, Kent SB. 1986. *J Biol Chem* **261**: 4229–4238.
4. Appel RD, Sanchez JC, Bairoch A, Golaz O, Miu M, Vargas JR, Hochstrasser DF. 1993. *Electrophoresis* **14**: 1232–1238.
5. Blattner FR, Plunkett G III, Bloch CA, Perna NT, Burland V, Riley M, Collado-Vides J, Glasner JD, Rode CK, Mayhew GF, Gregor J, Davis NW, Kirkpatrick HA, Goeden MA, Rose DJ, Mau B, Shao Y. 1997. *Science* **277**: 1453–1474.
6. Celis J, Gromov P, Ostergaard M, Madsen P, Honore B, Dejgaard K, Olsen E, Vorum H, Kristensen DB, Gromova I, Haunso A, Van Damme J, Puype M, Vandekerckhove J, Rasmussen HH. 1996. *FEBS Lett* **398**: 129–134.
7. Consortium TCeS. 1998. *Science* **282**: 2012–2018.
8. Cordwell SJ, Basseal DJ, Humphery-Smith I. 1997. *Electrophoresis* **18**: 1335–1346.
9. Di Luccia A, Iannibelli L, Addato E, Masala B, Manca L, Ferrara L. 1991. *Comp Biochem Physiol B* **99**: 887–892.
10. Eberini I, Miller I, Gemeiner M, Haynes P, Aebersold R, Puglisi L, Sirtori CR, Gianazza E. 1999. *Electrophoresis* **20**: 3599–3602.
11. Eng J, McCormack AL, Yates JR III. 1994. *J Am Mass Spectrom* **5**: 976–989.
12. Esteve-Romero JS, Yman IM, Bossi A, Righetti PG. 1996. *Electrophoresis* **17**: 1380–1385.
13. Fleischmann RD, Adams MD, White O, Clayton RA, Kirkness EF, Kerlavage AR, Bult CJ, Tomb J-F, Dougherty BA, Merrick JM, McKenney K, Sutton G, FitzHugh W, Fields C, Gocayne JD, Scott J, Shirley R, Liu L-I, Glodek A, Kelley JM, Weidman JF, Phillips CA, Spriggs T, Hedblom E, Cotton MD, Utterback TR, Hanna NC, Nguyen DT, Saudek DM, Brandon RC, Fine LD, Fritchman JL, Fuhrmann JL, Geoghagen NSM, Gnehm CL, McDonald LA, Small KV, Fraser CM, Smith CO, Venter JC. 1995. *Science* **269**: 496–512.
14. Goffeau A, Barrell BG, Bussey H, Davis RW, Dujon B, Feldmann H, Galibert F, Hoheisel JD, Jacq C, Johnston M, Louis EJ, Mewes HW, Murakami Y, Philippsen P, Tettelin H, Oliver SG. 1996. *Science* **274**: 546.
15. Gorg A, Postel W, Gunther S. 1988. *Electrophoresis* **9**: 531–546.
16. Guerreiro N, Redmond JW, Rolfe BG, Djordjevic MA. 1997. *Mol Plant Microbe Interact* **10**: 506–516.
17. Gygi SP, Rist B, Gerber SA, Turecek F, Gelb MH, Aebersold R. 1999. *Nature Biotechnol* **17**: 994–999.
18. Gygi SP, Rochon Y, Franza BR, Aebersold R. 1999. *MolCell Biol* **19**: 1720–1730.
19. Haynes P, Miller I, Aebersold R, Gemeiner M, Eberini I, Rosa Lovati M, Manzoni C, Vignati M, Gianazza E. 1998. *Electrophoresis* **19**: 1484–1492.
20. Hodges PE, Payne WE, Garrels JI. 1998. *Nucleic Acids Res* **26**: 68–72.
21. Honore B, Leffers H, Madsen P, Celis JE. 1993. *Eur J Biochem* **218**: 421–430.
22. Klose J, Kobalz U. 1995. *Electrophoresis* **16**: 1034–1059.
23. Lashkari DA, DeRisi JL, McCusker JH, Namath AF, Gentile C, Hwang SY, Brown PO, Davis RW. 1997. *Proc Natl Acad Sci U S A* **94**: 13057–13062.
24. Liang P, Pardee AB. 1992. *Science* **257**: 967–971.
25. Link AJ, Eng J, Schieltz DM, Carmack E, Mize GJ, Morris DR, Garvik BM, Yates JR III. 1999. *Nature Biotechnol* **17**: 676–682.
26. Link AJ, Hays LG, Carmack EB, Yates JR III. 1997. *Electrophoresis* **18**: 1314–1334.
27. Mann M, Wilm M. 1994. *Anal Chem* **66**: 4390–4399.
28. Matsudaira P. 1987. *J Biol Chem* **262**: 10035–10038.
29. Miller I, Haynes P, Gemeiner M, Aebersold R, Eberini I,

Copyright © 2000 John Wiley & Sons, Ltd. 

Rosa Lovati M, Manzoni C, Vignati M, Gianazza E. 1998. *Electrophoresis* **19**: 1493–1500.
30. Molloy MP, Herbert BR, Walsh BJ, Tyler MI, Traini M, Sanchez JC, Hochstrasser DF, Williams KL, Gooley AA. 1998. *Electrophoresis* **19**: 837–844.
31. Molloy MP, Herbert BR, Williams KL, Gooley AA. 1999. *Electrophoresis* **20**: 701–704.
32. O'Connor CD, Farris M, Fowler R, Qi SY. 1997. *Electrophoresis* **18**: 1483–1490.
33. Oda Y, Huang K, Cross FR, Cowburn D, Chait BT. 1999. *Proc Natl Acad Sci U S A* **96**: 6591–6596.
34. Opiteck GJ, Lewis KC, Jorgenson JW, Anderegg RJ. 1997. *Anal Chem* **69**: 1518–1524.
35. Opiteck GJ, Ramirez SM, Jorgenson JW, Moseley MA III. 1998. *Anal Biochem* **258**: 349–361.
36. Rosenfeld J, Capdevielle J, Guillemot JC, Ferrara P. 1992. *Anal Biochem* **203**: 173–179.
37. Santoni V, Doumas P, Rouquie D, Mansion M, Rabilloud T, Rossignol M. 1999. *Biochimie* **81**: 655–661.
38. Santoni V, Rabilloud T, Doumas P, Rouquie D, Mansion M, Kieffer S, Garin J, Rossignol M. 1999. *Electrophoresis* **20**: 705–711.
39. Sazuka T, Ohara O. 1997. *Electrophoresis* **18**: 1252–1258.
40. Service F. 2000. *Science* **287**: 2136–2138.
41. Shalon D, Smith SJ, Brown PO. 1996. *Genome Res* **6**: 639–645.
42. Shevchenko A, Chernushevich I, Ens W, Standing KG, Thomson B, Wilm M, Mann M. 1997. *Rapid Commun Mass Spectrom* **11**: 1015–1024.
43. Smaglik P. 2000. *Nature* **404**: 691–692.
44. Urquhart BL, Atsalos TE, Roach D, Basseal DJ, Bjellqvist B, Britton WL, Humphery-Smith I. 1997. *Electrophoresis* **18**: 1384–1392.
45. VanBogelen RA, Abshire KZ, Moldover B, Olson ER, Neidhardt FC. 1997. *Electrophoresis* **18**: 1243–1251.
46. Velculescu VE, Zhang L, Vogelstein B, Kinzler KW. 1995. *Science* **270**: 484–487.
47. Velculescu VE, Zhang L, Zhou W, Vogelstein J, Basrai MA, Bassett DE, Hieter P, Vogelstein B, Kinzler KW. 1997. *Cell* **88**: 243–251.
48. Wasinger VC, Bjellqvist B, Humphery-Smith I. 1997. *Electrophoresis* **18**: 1373–1383.
49. Wilkins MR, Pasquali C, Appel RD, Ou K, Golaz O, Sanchez J-C, Yan JX, Gooley AA, Hughes G, Humphery-Smith I, Williams KL, Hochstrasser D. 1996. *Bio/Technology* **14**: 61–65.
50. Yan JX, Tonella L, Sanchez JC, Wilkins MR, Packer NH, Gooley AA, Hochstrasser DF, Williams KL. 1997. *Electrophoresis* **18**: 491–497.
51. Yates JR III, Eng JK, McCormack AL, Schieltz D. 1995. *Anal Chem* **67**: 1426–1436.

*The* **Genomics** *Website at Wiley*

**www.wiley.co.uk/genomics**

The **Genomics** website at Wiley is a new and **DYNAMIC** resource for the genomics community, offering **FREE** special feature articles and new information **EACH MONTH**.

Find out more about *Comparative and Functional Genomics*, and how to view all articles published this year **FREE OF CHARGE**!

Visit the **Library** for hot books in Genomics, Bioinformatics, Molecular Genetics and more.

Click on **Primary Research** for information on all our up-to-the minute journals, including: *Genesis*, *Bioessays, Gene Function and Disease*, and the *Journal of Gene Medicine.*

Let the **Genomics** website at Wiley be your **guide** to genomics-related web sites, manufacturers and suppliers, and a calendar of conferences.

WILEY

1516

Copyright © 2000 John Wiley & Sons, Ltd. 
